# Supplementary material for: Non-Renal Risk Factors for Chronic Kidney Disease in Liver Recipients with Functionally Intact Kidneys at 1 Month
Source: J Clin Med. 2022 Jul 20;11(14):4203. doi: 10.3390/jcm11144203 (PMC9315935; doi:10.3390/jcm11144203)

Figure S1. Graphic depiction of matching process according to presence or absence of post-transplant risk factors.

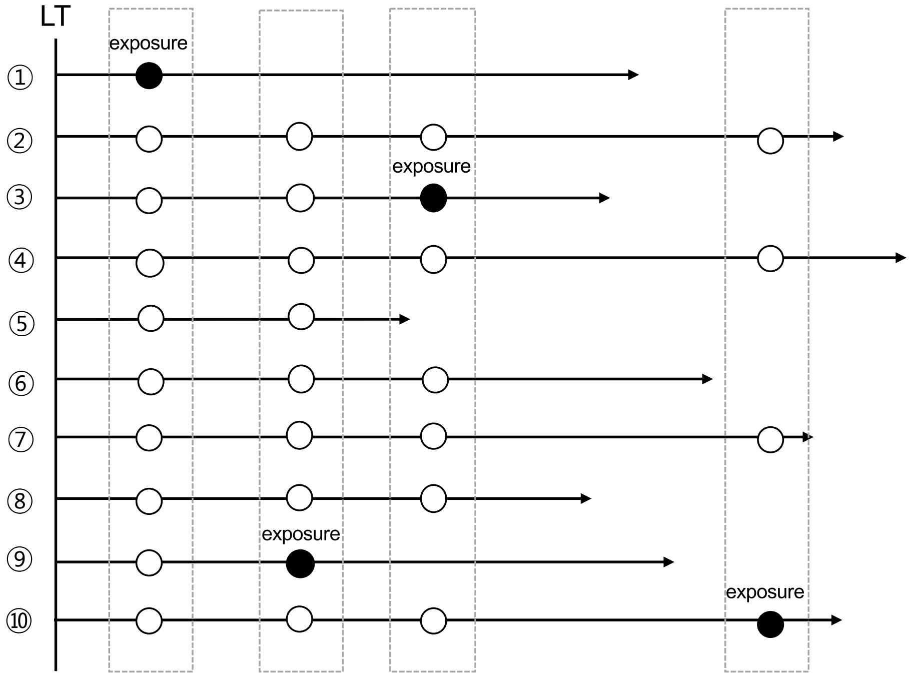

Black round means the first exposure to post-transplant risk factors in given patients. White round means potential control subjects for matching. Patients exposed to risk factors were matched to controls at 1:3 ratio on time-conditional propensity scores which was generated with baseline- and time-varying variables measured at each time points. From chronological matching process, patients selected as control group once were not considered as potential controls in further matching but were used again as risk group if appropriate.

LT, liver transplantation

Figure S2. Balance testing of propensity score-matched post-transplant risk factors.

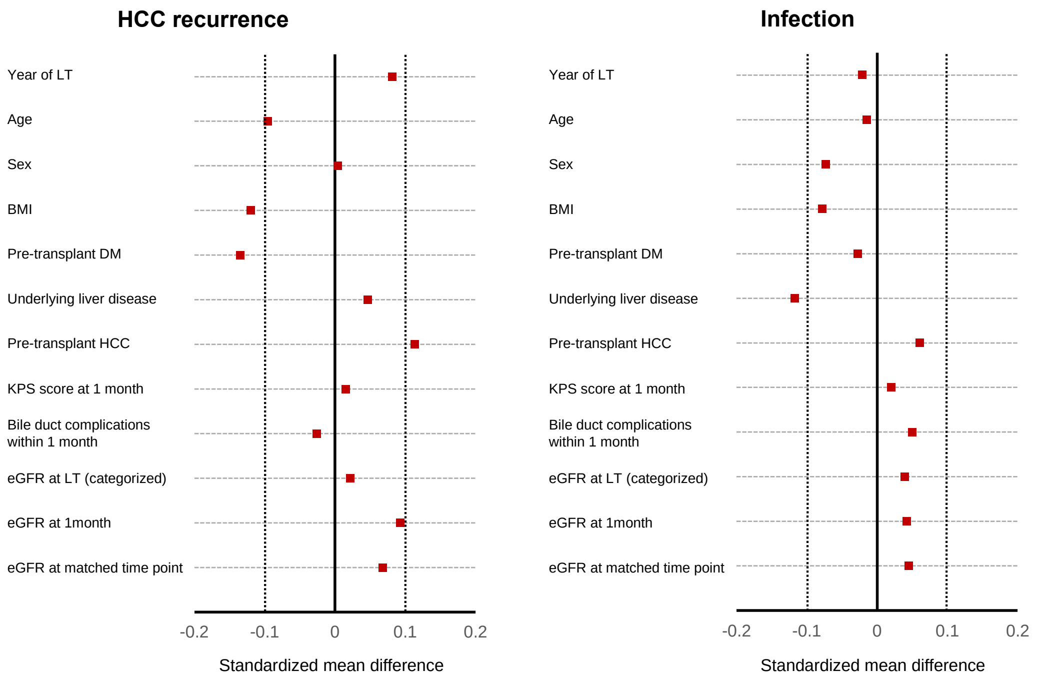

Standardized mean differences between -0.2 and 0.2 indicated adequate matching.

BMI, body mass index; DM, diabetes mellitus; HCC, hepatocellular carcinoma; KPS, Karnofsky performance status score; LT, liver transplantation

Table S1. Comparison of liver function 6 to 12 months after matched time points according to presence or absence of exposures.

Variables	HCC recurrence (n=137)	No HCC recurrence (n=383)	*P*
AST, U/L	32 (24-53)	24 (19-33)	<0.01
ALT, U/L	25 (17-44)	20 (14-34)	<0.01
INR	1.0 (0.9-1.1)	1.0 (0.9-1.1)	0.51
Total bilirubin, md/dL	0.7 (0.5-0.9)	0.7 (0.5-1.0)	0.12
**Variables**	**Infection (n=369)**	**No infection (n=1073)**	***P***
AST, U/L	28 (20-42)	23 (18-33)	<0.01
ALT, U/L	22 (13-43.8)	19 (13-32)	<0.01
INR	1.0 (0.9-1.1)	1.0 (0.9-1.1)	0.66
Total bilirubin, md/dL	0.7 (0.5-1.1)	0.7 (0.5-0.9)	0.04

Data expressed as median (IQR) because variables were not normally distributed.

ALT, alanine aminotransferases; AST, aspartate aminotransferases; HCC, hepatocellular carcinoma; INR international normalized ratio; TAC, tacrolimus

Table S2. Baseline characteristics, postoperative events and immunosuppressants according to recurrence of HCC.

Variables	No HCC recurrence (n=1001)	HCC recurrence (n=150)	*P*
Age, years	56.0 (51.0-61.0)	55.0 (49.0-59.0)	0.004
Sex, female	175 (17.5)	15 (10.0)	0.029
BMI, kg/m^2	24.2 (22.2-26.4)	23.5 (21.8-25.6)	0.016
Donor type			0.392
Living	913 (91.2)	133 (88.7)	
Deceased	88 (8.8)	17 (11.3)	
Donor age, years	30.0 (23.0-39.0)	29.0 (23.0-42.0)	0.900
Donor sex, female	356 (35.6)	60 (40.0)	0.335
ABO incompatibility	216 (21.6)	38 (25.3)	0.353

Hypertension	183 (18.3)	33 (22.0)	0.329
Pre-transplant DM	266 (26.6)	28 (18.7)	0.049
MELD	10.0 (8.0-14.0)	10.0 (8.0-13.0)	0.406
KPS at 1 month			0.560
High (80-100%)	405 (40.5)	55 (36.7)	
Intermediate (50-70%)	527 (52.6)	86 (57.3)	
Low (0-40%)	69 (6.9)	9 (6.0)	
Basiliximab induction	904 (90.3)	139 (92.7)	0.439
Immunosuppressants at 1 month			0.000
Tacrolimus + Mycophenolate mofetil	803 (80.2)	109 (73.2)	
Tacrolimus	177 (17.7)	28 (18.8)	
Others	21 (2.1)	12 (8.1)	
Tacrolimus trough level			
1 month	7.5 (5.7-9.6)	7.3 (5.5-10.1)	0.558
6 months	6.7 (5.1-8.5)	5.6 (4.0-7.4)	0.000
12 months	5.5 (4.3-7.3)	3.8 (2.2-6.0)	0.000
Biopsy-proven rejection	81 (8.1)	15 (10.0)	0.529
Bile duct complication	51 (5.1)	5 (3.3)	0.464
Vascular complication	322 (32.2)	53 (35.3)	0.498
Infection	80 (8.0)	15 (10.0)	0.500

Figure S3. Distribution of tacrolimus trough level.

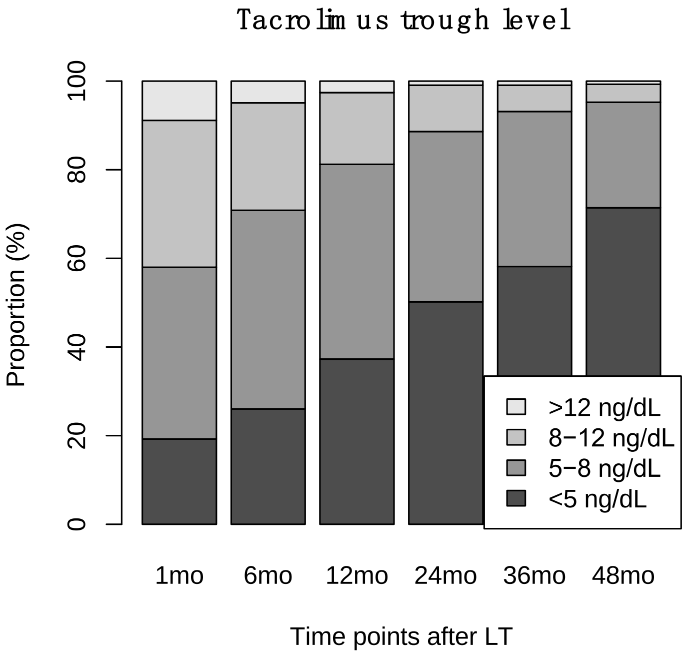

Supplement: Supplementary file 1 [file jcm-11-04203-s001.zip › jcm-1818237-supplementary.pdf]
